# Conducting a child injury prevention RCT in the wake of COVID-19: lessons learned for virtual human subjects research

**DOI:** 10.3389/fdgth.2023.1198314

**Published:** 2023-09-08

**Authors:** Sophia Prokos, Amy Damashek, Barbara Morrongiello, Emilie Arbour, Bethelhem Belachew, Farzana Zafreen

**Affiliations:** ^1^ Department of Psychology, Western Michigan University, Kalamazoo, MI, United States; ^2^ Department of Psychology, University of Guelph, Guelph, ON, Canada

**Keywords:** prevention, child injury prevention, child injuries, COVID-19, virtual data collection

## Abstract

Unintentional injury is the leading cause of death among children in the United States, and children living in low-income households are particularly at risk for sustaining unintentional injuries. Close parental supervision has been found to reduce young children's risk for injury; however, few studies have examined interventions to increase parental supervision. This paper discusses COVID-19 related modifications that were made to a federally funded randomized controlled trial to reduce low-income children's risk for unintentional injury. The study's procedures (data collection and intervention delivery) had to be transitioned from in-person to a fully virtual format. Modifications that were made to the study included use of: participant cell phones to conduct data collection and intervention sessions; virtual meeting software to conduct sessions with participants and; an online platform to collect questionnaire data. In addition, many modifications were required to complete the in-home observation virtually. In terms of feasibility, the investigators were able to collect all of the data that was originally proposed; however, recruitment and retention was more challenging than anticipated. Lessons learned during the modification process are included to provide guidance to researchers seeking to conduct virtual human subjects research in the future.

## Introduction

The impact of the coronavirus (COVID-19) pandemic on human subjects research has been substantial. Many studies were either paused or required significant modification due to institutional regulations or difficulty conducting research amid concerns about transmission of the virus (1–3). This article will discuss modifications that were made to an NIH (National Institutes of Health)—funded child injury prevention study (1R15HD097585-01A1) in the wake of COVID-19. Given that data collection had to pivot from in-person (in participants' homes) to virtual, lessons learned from this study may be applicable to other investigators attempting to conduct human subjects research virtually. The present paper will provide a brief overview of the project, followed by a discussion of the modifications that were made to the study, and lessons learned. Data will also be presented about study feasibility. The paper is not intended to report the results of a randomized clinical trial but to provide guidance for researchers attempting to conduct virtual human subjects research.

## Brief overview of study rationale

This study aimed to modify and assess the efficacy of a child injury prevention intervention (Supervising for Home Safety) for a low-income population in a mid-sized midwestern community. Unintentional childhood injuries are a serious public health problem in the United States and are the most common cause of death for children ages 1–19 (4). Moreover, several studies have found that children living in low-income households are at an increased risk of sustaining unintentional injuries compared to their higher income peers (5–8). For instance, research has found that low-income families frequently lack safety devices for their homes or cannot afford good quality childcare to supervise their children effectively. Moreover, substandard housing can also increase children's risk for injury (9–11).

Several studies have found that close parental supervision can reduce the frequency and severity of childhood injuries (12, 13), suggesting that effective interventions to increase parental supervision are needed. One intervention (Supervising for Home Safety), has been found to increase parental supervision of young children, however, it has only been tested among middle-upper income families in Canada (14, 15). Thus, the aims of the present study were to modify the Supervising for Home Safety intervention for low-income U.S. families and to test the efficacy of the modified intervention in increasing parent supervision and reducing children's injury frequency. The creator of this intervention (Barbara Morrongiello, a co-investigator on the grant) collaborated on the creation of the modified intervention materials and on developing procedures for the observation; however, she was not actively involved in the RCT data collection process.

Prior to conducting the RCT, two rounds of focus groups were conducted with parents of children enrolled in preschool programs for low-income families. Data from the focus groups were used to identify modifications that needed to be made to the intervention to make it more appropriate for low-income U.S. families. The intervention materials were then modified based on focus group feedback. Modifications included recording new program videos with local low-income families who were more racially and ethnically diverse than the families who were depicted in the original program videos. Program handouts were also modified to make them more user friendly [see (16) for more detail]. The RCT examined the modified intervention's impact on parents' supervision practices and children's injury frequency. The first round of focus groups occurred prior to the onset of the COVID-19 pandemic and were conducted in person. The second round of focus groups occurred during the pandemic and were conducted virtually. Participants joined the virtual focus groups via computer or cell phone using a Webex link, and the focus group was recorded via Webex.

## Summary of study methods

### Recruitment

Parents were recruited for the RCT from three local preschool programs that serve low-income families. Specifically, parents were recruited from two Head Start programs (one program was located in the community in which the study was taking place, and one was located in an adjacent community) as well as an additional program that provides discounted preschool to low-income families. Parents were eligible for the three local preschool programs if their income fell at or below 100% of the U.S. federal poverty level (see https://www.healthcare.gov/glossary/federal-poverty-level-fpl/ for more information). Participants were recruited via referral from program home visitors as well as dissemination of flyers via email and social media. The preschool programs were voluntary, and participants were notified that their participation in the study was voluntary as well. They were also notified that their decision about whether or not to participate would not affect the preschool services that they were receiving.

Participants were compensated for their participation and could earn between $170 and $270 in gift cards, depending on whether they were in the control or treatment group. Participants were also provided with prepaid cellphones and data to participate in data collection. Instructions about how to use the cellphones were also provided. This study was approved by the Institutional Review Board at the primary investigator's university. All participants provided informed consent electronically before participating in the study. The RCT was registered with clinicaltrials.gov (protocol id# AD3874920).

### Intervention summary

The Supervising for Home Safety program was designed to be delivered individually to participants in 1 h weekly sessions for five weeks. For each of the five weeks, parents participate in video-based education about child injury prevention. In session 1, parents watch and then discuss a 20 min introductory video that is designed to increase parents' awareness of their child's risk for unintentional injury and the importance of supervision. In subsequent sessions, parents watch video vignettes depicting common at-home injury scenarios in several injury categories (e.g., burns, falls, poisonings). Parents are also introduced to a problem solving approach (ALTER) to learn about strategies to reduce their child's risk for injury (e.g., change the child's location, change the environment, use resources). Parents practice applying the ALTER strategies to the video vignettes in session and also practice applying ALTER at home between intervention sessions.

### Measurement

Data for the RCT were collected via questionnaire measures, parent–child observations, and weekly injury interviews.

#### Questionnaire data

A set of questionnaires were administered at pre-test, post-test and at 1 month follow-up to assess parents' supervision practices, readiness for changing supervision practices, and injury prevention beliefs. Additional questionnaires were administered at pre-test to assess demographics and family risk factors (e.g., parent depression, substance use, domestic violence). Finally, two additional questionnaires were administered at post-test to assess participant satisfaction with the intervention.

#### Observational data

Parent–child observations that were 20 min in length were conducted at pre-test, post-test, and follow-up to gather an objective measure of parents’ supervision of their young children in an injury risk situation. A “Gadget” (a contrived pseudo hazard) was set up in participants' homes to stimulate a situation in which the parent had to supervise the child in the presence of a hazard (17). Video recordings were coded to assess for the parent's supervisory behaviors and the child's risk behaviors.

#### Injury interviews

Children's minor injury frequency was assessed weekly via 5–10 min telephone interviews. Participants were provided with prepaid cell phones to participate in the weekly injury interviews.

## COVID-19 related barriers and modifications

Prior to the second round of focus groups and the RCT, due to the spread of the COVID-19 virus, the investigators' university suspended all research that involved any in-person contact. Investigators wishing to conduct research were required to submit a detailed research restart plan describing how they would modify study procedures to minimize the risk of COVID-19 transmission. The research restart plan for the present study included a switch from in-person to completely virtual operations and data collection. All staff meetings were conducted virtually via the video conferencing application WebEx. In addition, access to campus was restricted so that only the primary investigator had permission to access the lab and its materials during the early phases of the RCT. The modifications that were made to the study in order to comply with university COVID-19 protocols and to limit the spread of COVID-19 are described below. The paper will also include “lessons learned” that may be helpful for other researchers attempting to conduct fully virtual research with human subjects (see Table 1 For a summary of lessons learned).

**Table 1:** Study modifications and lessons learned*.*

Modifications	Lessons learned
Virtual data collection	Participants need access to electronic devices of sufficient quality and enough data that allow for video-chatting. Virtual data collection platforms can be used to collect questionnaire data.
Observation material drop-off and pick-up	Provide clear instructions about material drop-off and pick-up (e.g., safety protocols, estimated time of arrival). Provide a set of instructions for drivers and data collectors.
Cell phone and tripod setup	Provide step-by-step participant instructions for cellphone and tripod set up. Provide instructions for data collectors to prepare for difficulty with set up. Pilot test the positioning and set up of the tripod.
Cell phone audio issues	Purchase external microphone for higher quality audio.
Lighting challenges	Select a room with adequate lighting and use explicit instructions to aid participants in room selection. Provide lighting (i.e., spotlight) if necessary.
Connectivity issues	Have an alternate video meeting platform available. Check WIFI accessibility of participants and budget for more data for participants who may need it.
Gadget placement	Standardize procedures, make use of multimodal instructions, and pilot test procedures.
Engaging the child in the session	Provide toys or activities for children with explicit instructions about what the child should and should not play with during the observation.
Multiple children in the home	Assist the parent in coordinating child care or engaging their child in a safe activity while under the parent's supervision.
Tracfone difficulties	Use higher quality phones. Alternatively use tablets or laptops.
Length of the data collection session	Pilot test for the length of the session. Provide tablets for parents to complete questionnaires.
Data and material storage	Have protocols in place for remote data entry and storage. Use a tracking system to be able to track materials. Provide secure storage containers to all study personnel.
Fewer participants due to COVID-19	Have backup recruitment sites in mind and build connections with community organizations.

The following modifications are discussed below: general data collection; parent–child observations; length of data collection sessions; data and material storage; recruitment challenges, modifications to the intervention, and feasibility. Each section will be followed by lessons learned. Despite challenges related to virtual data collection, the investigators were able to retain all measures that were proposed in the research grant (as discussed above).

### General data collection modifications for the RCT

Study personnel had originally planned to conduct data collection in participants' homes for the pre, post, and follow-up data collection sessions. Instead, the video conferencing application WebEx was used to conduct the entirety of the data collection sessions. In order to make it feasible for participants to engage in virtual data collection sessions, some of the grant budget had to be reallocated for this purpose. The original grant budget included funds to provide prepaid cell phones for all participants to conduct the weekly injury interviews; however, to enable participants to engage in video chat meetings for the pre, post, and follow-up sessions, cell phone data plans also had to be purchased for each participant. Study personnel also had to pre-upload the WebEx software to the cell phones before distributing them to participants, in addition to the minutes and data. Finally, as detailed below, additional funds had to be reallocated to purchase lapel microphones to improve the sound quality of the observations.

Although the prepaid cell phones were used somewhat successfully, several participants encountered challenges to setting up the cell phones. Participants had difficulties locating the applications (WebEx) on the cell phones that were necessary to complete the data collection session. Furthermore, navigating the audio and visual features of the cell phones was also challenging. Thus, study staff created a set of instructions for parents to use in order to assist them in using the cellphone for the virtual meetings.

Another challenge was determining how to administer the questionnaires to the participants; in the original study protocol, questionnaire data were going to be collected via paper and pencil. All questionnaires were transferred from paper to Qualtrics, an online survey platform. Survey links were emailed to participants using a study email, and participants completed the online surveys using the study phone or their personal phone (the majority of participants did not have access to laptops or tablets). Successful survey completion was monitored online by study personnel. Participants also used Qualtrics to consent to the study. Participants received a link to the consent form, and the research staff reviewed it with them; participants indicated their consent to participate via Qualtrics.

#### Lessons learned

In order to conduct virtual data collection sessions, participants need access to electronic devices that allow them to engage in video chats and to complete questionnaires. When working with low-income populations in particular, it is necessary to provide data in addition to devices, for those who do not have access to the internet. This study provided Tracfones to the participants; however, these devices were not ideal due to their small size as well as their limited audio and picture quality. Tablets or inexpensive laptops would be more effective devices to loan to participants to enable better virtual meeting quality, if the study budget allows for it. Additionally, virtual data collection platforms are useful in disseminating surveys and/or questionnaires to participants. Platforms such as Qualtrics allow researchers to create links for their surveys and send them to participants via email while monitoring the progress and completion of each survey. Finally, it is helpful to create specific instructions for participants who may need assistance setting up their electronic devices.

### Parent–child observation

As noted above, the RCT included a 20 min recorded observation of parent–child interaction at pre, post and follow-up. The observations were originally intended to be conducted in-person in participants' homes. Conducting the observations virtually was the most difficult challenge that was encountered. Challenges fell into several categories, including: set up of observation materials; camera positioning; cell phone audio problems; lighting issues; Tracfone problems; connectivity issues; and difficulties managing child behavior.

#### Set-up of observation materials

Study personnel had originally planned to bring the observation materials to participants' homes and to set up the Gadget, as well as a videorecorder and tripod to record the parent–child observation. Instead of using a video camera, WebEx was used via participants' study phones to conduct and record the virtual observation. Participants were not asked to set up a video camera on their own because data collectors needed to be able to view the observation while it was occurring, to make sure the view from the recording device was adequate for coding purposes. Thus, participants placed their phones on the tripods while participating in a WebEx video chat session with the data collectors. The data collectors then recorded the video chat session via WebEx (by pressing the record button).

Determining a way to set up a recording device in participants' homes without entering their homes was a challenge. Data collection materials were dropped off inside a bin at the participant's doorstep to avoid any in-person contact. Drivers needed to follow safety protocols such as properly washing their hands before dropping off materials and sanitizing materials prior to drop off and after pick-up. Some participants allowed their child to see the Gadget prior to the observational period. The Gadget was intended to be a novel stimulus to the child during the observation, which would pique their curiosity so that they would be enticed to try to contact it. Thus, clear instructions were developed for the participants about when to open the bin and when to set up the materials for the observation.

##### Lessons learned

Conducting in-home observations virtually is a challenge. A great deal of planning and pilot testing is needed to make this process go smoothly. Clear and consistent communication with participants is critical when dropping off and picking up study materials. It is important to provide the participants with the relevant information and instructions needed to complete the drop-off and pick-up process (i.e., where and when the materials will be dropped off, when to put it back outside on their doorstep). It is also important to make it clear to participants when it is appropriate to remove the items from the bin and what to do with them. Finally, it is important to provide participants with clear instructions about how to set up the observational materials (i.e., cell phone and tripod).

#### Camera positioning

At the beginning of the study, data collectors instructed parents to set the prepaid phone on a flat and well-supported surface in order to position the phone in a way that data collectors could see a wide-angle view of the room. Prepaid cell phones were set up on the floor, on kitchen counters, and on coffee tables as surfaces. As one might suspect, this did not give an adequate view of the observation scenario. Data collectors were unable to see most of the room, the child, the parent, or the Gadget. In order to solve this issue, tripods were purchased that were designed to be used with cell phones. Parents were provided with instructions about how to set up the tripod and place the cellphone into the tripod. Use of the tripods allowed study staff to have more control over the positioning of the phone and its placement within the room and significantly improved the angles of the video recordings so that data collectors were better able to see the room, the child, the parent, and the Gadget. On occasion, participants experienced difficulty with setting up the tripod, despite the use of the instructions. For example, sometimes parts of the tripod did not function or maneuver properly. Due to these difficulties, a guide was created for the data collectors in order to assist the participants if issues with the tripod were to arise.

##### Lessons learned

Due to the fact that no data collectors were present in participants' homes, it was challenging to assist the participants when they encountered difficulty with the materials. Therefore, creating clear step-by-step instructions (with visuals included) for the participants was critical in allowing them to be successful in setting up the materials. In addition, it is important that the data collectors be prepared for these difficulties and have their own guide that they can use to assist the participants with cell phone and tripod set up.

#### Gadget placement

Another issue that was encountered was the placement of the Gadget in the room. The Gadget was to be placed in the room within the child's reach. Some parents placed the Gadget on the ground, some on tables, and others on countertops outside of the child's reach. A standardized setup was created so that all Gadgets were set up the same way with explicit instructions. The Gadget was to be placed on top of a milk crate (provided to participants) in the center of the room, near an outlet so that the lights could be plugged in.

##### Lessons learned

It is important to standardize the observation procedures, particularly for virtual data collection, when researchers cannot be present in the home to arrange materials. Multimodal instructions were helpful in setting up the materials with the participants, which included written instructions as well as a picture of what the material set-up should look like. It was also helpful to provide explicit instructions for the data collector to guide the participant in setting up the materials. Practicing and pilot testing the standardized set-up is critical in maintaining consistency across observations.

#### Cell phone audio issues

Although use of the tripod provided a wider vantage point in the room, data collectors encountered other video recording issues, including difficulty with audio. Study personnel had originally relied on the cell phones to record audio; however, the quality of the cellphones was limited and the microphones on the cellphones were not adequately picking up the audio. The tripod needed to be placed further away from the parent and child in order to achieve the wide vantage point of the room, which made it more difficult to record the parent's voice. To solve this issue, lapel microphones were added to the observations to enhance the audio recording; the parents were instructed to plug the lapel microphone into the headphone jack of the cell phone and to clip the microphone onto their clothing.

##### Lessons learned

The cell phones used for this study were not ideal in picking up participant audio during the data collection session. Using a higher quality cell phone or a tablet would have been beneficial and would likely improve the sound quality. Even with the use of higher quality cell phones or tablets, the use of an external microphone may be necessary to provide the best sound quality. Conducting a pilot test for the sound quality of cell phones or tablets, and the use of an external microphone is advised.

#### Lighting challenges

During many observations, data collectors encountered poor lighting issues. Some views of the parent and child were backlit by large windows with the sun shining into the room, making the participant a silhouette and not allowing the data collectors to see their faces. Furthermore, some participants set up the materials in poorly lit rooms with minimal to no light. Thus, specific instructions were developed in order to avoid these issues. Data collectors instructed participants to set up materials in a room with proper lighting and to turn on all lights, if possible. In addition, data collectors tried to ensure that the view of the cell phone camera was not backlit by large windows.

##### Lessons learned

Detecting the best location in the participant's home in order to maximize lighting can be challenging. It is important to include explicit instructions about lighting in the data collector guide that can be relayed to the participants before conducting the observation. It may also be useful to provide some form of lighting (e.g., spotlight or lamp) to the participants in case optimal lighting cannot be achieved with the participants' own household lights.

#### Tracfone difficulties

The investigators also experienced difficulties with the Tracfones. In some cases, the Tracfones would not function properly. Their video cameras would shut off unexpectedly, disrupting the observation. In other cases, navigating the Tracfones became difficult for participants as they were not used to the way that they function. This navigation became time consuming and caused frustration for some participants. In these cases, some participants preferred to use their personal phones due to familiarity and ease.

##### Lessons learned

Using higher quality phones or tablets would likely lead to fewer challenges with regard to functioning. Budgeting more for these higher quality items is recommended, if possible.

#### Connectivity issues

As one might expect, internet connectivity issues were also a frequent occurrence. In some instances the cell phones would not connect to in-home Wi-Fi properly. In other instances there was poor audio or video connection, or the WebEx application did not function properly. Study personnel had to problem solve on the spot in order to maintain network connectivity and rapport with the participant, as these difficulties would sometimes be accompanied by participant frustration. In addition, some participants did not have in-home Wi-Fi. For these participants, extra gigabytes of data were provided to use during the virtual data collection sessions.

##### Lessons learned

Some connectivity issues were challenging to resolve virtually. In some cases with poor audio connection, video connection, and WebEx application malfunction, data collectors were able to use an alternate video meeting application (Google Meet), and this resolved those issues. Having an alternate platform to use for virtual sessions in case any problems arise is advisable. In addition, it is important to check with participants regarding their Wi-Fi accessibility before giving them the study device so that extra data can be provided if needed. When planning a study, it is also important to budget for the extra data for participants who do not have access to their own Wi-Fi.

#### Managing child behavior during data collection

The original study protocol included the assistance of undergraduate research assistants and data collectors to help ensure that the child remained in the same room with their parent during the parent–child observation. This was not possible when conducting the observations virtually because there were no staff in the home to help the parent manage the child's behavior. In order to attempt to keep the child in the same room as their parent and in view of the camera, the children were provided with toys (e.g., crayons and coloring pages) to help keep them engaged in the observation session. If the child was uninterested in the toys that were provided, data collectors also encouraged parents to bring some of the child's favorite toys into the room. Tablets and the use of television was discouraged because they are so engaging that children would be less likely to attend to the Gadget.

Investigators had also originally planned for the undergraduate research assistants to supervise the participants' children when they were completing questionnaires (as well as during the intervention sessions). This was no longer an option with virtual data collection. Thus, data collectors assisted participants in finding alternative options to supervise their children when completing questionnaires. Some participants were able to ask family members and neighbors to assist them. For those participants who were unable to use those resources, the data collectors assisted the participants in finding a safe activity for their child to engage in while remaining in the same room and under the parent's supervision.

##### Lessons learned

It was helpful to provide an activity that children could engage in to keep them in the same room as the parent and within the frame of the observation. However, it is important to have explicit instructions about what type of materials they should not be engaging with during the observation (e.g., watching tv and playing games on tablets) to increase the likelihood that the child will engage with the parent or an item in the observation (e.g., a pseudo hazard). In addition, problem solving with the participant in advance about how children will be cared for during the data collection session is recommended. Assisting the parent in advance to locate childcare services (e.g., family or friends) or identify safe activities for the children to engage in (e.g., watching a movie while in the same room as the parent) would help to make data collection go more smoothly.

#### Overall lessons learned from the observation

Virtual in-home observations are possible, but challenging. It is important to create clear instructions for data collectors to assist participants in setting up the materials in their home. It is also important to provide clear instructions for participants, that includes information about the environment in which the observation should be conducted (e.g., living room area rather than bedroom, well-lit) and how to set up any materials that will be used for the observation. Using WebEx on the Tracfones worked sufficiently for this study; however, additional materials were necessary to improve the quality of the observations. External microphones significantly improved the audio quality during observations. In addition, the use of a tripod is necessary in order to provide a stable setting for the phone and to provide a wide-angle view of the environment. In this study, modifications were made throughout data collection and required a lot of trial and error. A pilot test period is helpful in order to identify possible challenges before they arise during data collection; however, researchers may need to continue to problem solve beyond the pilot test period. In addition, it is important to watch the observation recordings as they are collected in order to identify problems of which data collectors may be unaware (e.g., poor audio quality).

### Length of data collection sessions

The original in-home data collection sessions were intended to last approximately 1 h. However, the virtual data collection session lasted closer to 2 h. The additional instructions for setting up the observation materials (e.g., Tracfone, tripod, Gadget) and assisting the parent in problem-solving how to manage multiple children during the data collection sessions extended the duration. In addition, originally parents were to complete paper forms of the questionnaires during the observation. However, with the modifications, they had to complete the questionnaires online. Since they needed to use their cell phones to record the observation sessions, they had to complete the questionnaires after completing the observation, which extended the session duration. Due to the many frustrations with the Tracfones, difficulties with setting up the observation materials, and the extended length of the data collection sessions, data collectors experienced increased difficulty with rapport between the data collectors and the participants. This may have contributed to increased study attrition.

#### Lessons learned

Study personnel did not expect the length of the data collection session to increase as much as it did once all data collection procedures went virtual. It is important to pilot test for the true length of the session. In addition, reducing the amount of questionnaires or shortening the length of the observation may also be needed to reduce the length of the data collection sessions, thereby reducing participant burden. Completing the questionnaires on the Tracfones was not ideal given the smaller screen, making it more challenging to see the questions and select responses. It would be useful to provide tablets instead, which could be returned after completion of each data collection session.

### Data and material storage

The study's research restart procedures at the beginning of the pandemic severely limited study personnel's' access to the lab on campus; thus, data and materials were temporarily stored in the homes of study personnel (which was approved by the IRB). Locking filing cabinets and lockboxes were purchased in order to keep participant data secure. Graduate research assistants traveled to undergraduate research assistant homes weekly to pick up data. The graduate research assistants then would drop off the data to the home of the primary investigator who then took it to the lab on campus. Data entry was also completed remotely from undergraduate research assistants' homes. This required the undergraduates to gain remote access to the university server. Inevitably, study personnel encountered disruptions to picking up data from undergraduates' homes and delivering it to the primary investigator's home due to positive COVID-19 cases among study personnel. In addition, materials such as gift cards and cell phones were stored in the data collector's homes, which created difficulty in tracking study materials.

#### Lessons learned

It is critical that the proper protocols are in place when it comes to remote data entry and storage. It is ideal to keep all of the data in one designated location, if possible. If needing to distribute materials throughout the homes of multiple study personnel, it is recommended that a tracking system is created and used consistently in order to adequately track all materials. Moreover, it is important that secure storage containers are provided if research assistants are temporarily storing data in their homes.

### Recruitment challenges due to COVID-19

In addition to the previous issues, the researchers confronted challenges in recruiting participants because there was a lower participant pool due to COVID-19. For instance, for one of the Head Start locations, half as many children enrolled in the program for the first year of our recruitment. In order to address this challenge, recruitment efforts were expanded to a Head Start in an adjacent county (50 miles away). This required hiring research assistants in this new area to transport materials to and from the participant's homes. Moreover, more materials needed to be created and delivered to these research assistants. Nevertheless, recruitment was still slower than what was anticipated by the researchers. In addition to lower enrollment numbers in the preschool programs, it was also difficult to recruit participants as they were tired, “burned out”, and overwhelmed during the COVID-19 pandemic.

#### Lessons learned

It is worthwhile to anticipate potential recruitment shortfalls in advance and have backup plans prepared for additional recruitment sites. Developing strong relationship with community organizations helps to create opportunities for potential research partnerships in the future.

### Challenges delivering the intervention

Intervention sessions were provided by graduate research assistants who used a manual that provided detailed instructions for each session. Intervention sessions were originally designed to be delivered in participants' homes; however, these sessions were delivered virtually as well via WebEx. This posed some challenges, such as distractions during the sessions and technical difficulties. On occasion, parents needed to tend to children in the home or engage in household activities during the sessions. Moreover, on some occasions, participants would juggle their multiple responsibilities by engaging in sessions while in their car (although not while driving).

In addition, the video-based education for the intervention was originally planned to be presented to the parent in their home via a study computer. Videos now needed to be shared via the WebEx meetings. This was done by sharing the research assistant's screen in order for the participant to see the video. Screen sharing was also used to review program handouts. Participants were provided with printed handouts in advance that they could refer to as well. When sharing the screen, some participants had difficulty both seeing and hearing the videos. In these cases, additional time was added to the session to troubleshoot the visual and audio issues. On some occasions, the session needed to be transferred to a different online video meeting platform (i.e., Google Meet).

#### Lessons learned

When completing intervention sessions virtually, it is important to coordinate with the participant and plan accordingly if there are multiple children in the home. It would be ideal to schedule intervention sessions during a time when there are no children home or when there is another adult in the home who can supervise the children. This way it is possible to maintain the parent's sustained attention without environmental distractions. If these options are not possible, it is important to assist the parent in finding a way to safely keep the child/ren occupied and in view during the session. In addition, it is helpful to have an alternative video meeting platform in case the primary platform is not functioning properly.

## Feasibility

Despite modifications that were made to the protocol to collect data virtually, the investigators were still able to collect all of the data that was proposed in the original study protocol (i.e., questionnaire measures, phone interview data, and observations). However, difficulties were encountered with regard to meeting the study's recruitment and enrollment goals. Recruitment is ongoing at this time, but as of March 2023, study staff received 248 referrals from the three programs. Of those referrals, 29% declined to participate in the study. Common reasons for declining to participate included parent's time constraints, concerns with the recording of the in-home observation, and family health concerns. Approximately, 29% were lost to contact after study staff actively attempted to recruit them for a period of two months. The remainder (104, 42% of referrals) indicated that they were interested in participating after an initial recruitment phone call. Of those parents who indicated interest after the recruitment phone call, 80 participants were consented. Of those who were consented, 61% completed their post-test data collection sessions, resulting in an attrition rate of 39%. This attrition rate is higher than the anticipated attrition rate of 25%. It is possible that several of the challenges that were mentioned above may be responsible for a higher attrition rate, including prolonged data collection sessions, technological difficulties, less support for participants during data collection and intervention sessions, and participants' general fatigue as a result of dealing with the pandemic. Given the multiple challenges that participants faced, one might surmise that parents who completed the study were highly motivated, which may be related to the study incentives or participant interest in the study topic.

### Lessons learned

Multiple methods can be used in future virtual research studies in order to reduce attrition rates. One method that can be used is calling participants frequently to remind them of appointments to help ensure that the appointments occur as scheduled. Sending letters to participants could also be of use for those participants who are difficult to reach via phone, text, or email. In addition, reducing the demands of the data collection sessions through shortening the sessions or reducing the amount of questionnaires could reduce participant burden and frustration with data collection procedures.

## Conclusion

Transitioning a human subjects research project from in-person to completely virtual amid COVID-19 has been possible but challenging. It is difficult to know exactly how much COVID-19 affected the study's recruitment and retention rates. However, the experiences of the research team resulted in potentially helpful suggestions for researchers who wish to conduct virtual data collection in the future (Table 1). Useful tools for virtual data collection (see Table 2) include online video chat platforms (e.g., WebEx, Google Meet), as well as secure survey platforms (e.g., Qualtrics). Virtual observations present many challenges and require a great deal of planning and trouble shooting. Some tools that were identified as necessary include tripods and lapel microphones. It is also important to provide clear instructions for both the data collectors and the participants about how to set up the observation setting and materials. Multimodal instructions can be helpful, including the use of visuals. Finally, particularly when working with low-income individuals, provision of devices is necessary, and provision of Wi-Fi may also be needed. Budgeting for good quality devices (e.g., tablets), makes data collection easier for participants, and may improve the quality of observational data.

**Table 2:** Digital modifications.

Modifications	Purpose
Qualtrics	Qualtrics is a virtual data collection platform that allows for surveys to be digitally distributed to participants via a link. The data from these surveys are also easily accessible on this platform
WebEx	WebEx is one of many virtual meeting applications that allows for video chatting with participants for an unlimited amount of time.
Prepaid cell phones	Cell phones can be distributed to participants to use for virtual meetings, text messaging, and phone calls.
Lapel microphones	External microphones can be purchased to enhance audio of recordings

## Data Availability

The datasets presented in this article are not readily available because the data cannot be shared. Requests to access the datasets should be directed to AD, Damashek@wmich.edu.
